# Sulfur mustard induced oxidative stress and its alteration using asoxime (HI-6)

**DOI:** 10.2478/intox-2013-0029

**Published:** 2013-12

**Authors:** Miroslav Pohanka, Jakub Sobotka, Hana Svobodova, Rudolf Stetina

**Affiliations:** Faculty of Military Health Sciences, University of Defense, Trebesska 1575, 50001 Hradec Kralove, Czech Republic

**Keywords:** blister agent, acetylcholinesterase, oxidative insult, reactive oxygen species, cholinergic anti-inflammatory pathway

## Abstract

Sulfur mustard (SM) is a blister agent with cytotoxic mechanism of action. There is no suitable treatment based on administration of an antidote. In this study, Wistar rats were exposed to SM in doses of 0–40 mg/kg body weight and treated with the compound HI-6. The treatment provided no significant effect on ferric reducing antioxidant power of blood and plasma. However, HI-6 caused an increase in the level of thiobarbituric acid reactive substances. This stressogenic response was presumably the cause of the significant elevation of the blood level of both glutathione reductase and reduced glutathione. HI-6 appears to be suitable for enhancing prophylactically oxidative stress protection from small oxidative insult.

## Introduction

Sulfur mustard (SM), bis(2-chloroethyl)sulfide (CAS: 505-60-2), is one of the most important chemical warfare agents. Due to its physiological action, it is marked as a blister agent. SM acts as a cytotoxic agent with detrimental effect on brain, kidney, muscles, spleen, liver, marrow and other organs (Paromov *et al.*, 2007). The most serious SM molecular impact is DNA damage followed by over-stimulation of poly(ADP-ribose) polymerase (PARP). It leads to exhaustion of cell energy and uncovered generation of reactive oxygen species (ROS) with detrimental consequences (Korkmaz *et al.*, 2008a). The role of oxidative stress was recognized also in Iranian victims of the Iran-Iraq war who were seriously poisoned with SM (Shohrati *et al.*, 2008, 2009). They found a significant decrease of low molecular weight antioxidants and alteration in the activities of some enzymatic antioxidants.

Regarding oxidative stress accompanying SM exposure, application of exogenous low molecular weight antioxidants can be a logical measure in SM therapy. Experiments have provided some support for the suitability of antioxidant therapy. Some individual antioxidants, such as melatonin, were recommended to deserve higher attention (Korkmaz *et al.*, 2009b). The expected mitigating effect of antioxidants has however not unambiguously been established. Thus e.g. application of epigallocatechin gallate (EGCG) was not found to exert beneficial effect on total blood level of antioxidants or protection from lipoperoxidation. On the contrary, some adverse effects related to oxidative stress were observed (Pohanka *et al.*, 2011a). Hypothetically, we suggest another way how to decrease oxidative stress after SM poisoning. In our previous research, we found a slight temporary rise of oxidative stress after application of asoxime (HI-6) to laboratory animals (Pohanka *et al.*, 2010). In this work, we attempted to induce antioxidant self-defense by application of HI-6 prior to SM poisoning and thus to reduce the ensuing oxidative stress. Due to the limited efficacy of antioxidant therapy, the experiment proposes an alternative approach to therapy. The enhanced antioxidant barrier can be a potent protection against the detrimental effects of SM.

## Material and methods

### Animal exposure and blood sampling

Female Wistar rats (n=54) weighing from 180 to 200 g were purchased from Velaz corp. (Prague, Czech Republic). The whole experiment was supervised by the Ethic Committee of the Faculty of Military Health Sciences, University of Defence, Hradec Kralove, Czech Republic and the animals were manipulated in compliance with permission. For the whole experiment, the animals were kept in an airconditioned room (temperature 22±2 °C; humidity 50±10%; light period 12 h per day). The animals had full access to food and water.

Pure distilled SM was achieved from the Military Technical Institute of the former Czechoslovak Army and manipulation was permitted by the Czech Republic Governmental Institution SUJB, the official representative of the Organization for Prohibition of Chemical Weapons (OPCW) in the Czech Republic. SM was applied on a closely clipped intercapular region. HI-6 (asoxime chloride; CAS 34433-31-3; ((2-hydroxyiminomethylpyridiniummethyl)-(4’-carbamoylpyridiniummethyl))ether dichloride) was administered intramuscularly in doses of 2 or 20% LD_50,_ corresponding to 15.6 and 156 mg/kg b.w.

The animals were divided into nine groups, by six animals each. HI-6 was applied 15 minutes prior to SM. The groups were exposed as follows: 1) control: intramuscular and dermal application of saline solution; 2) 15.6 mg/kg of HI-6, saline solution dermal; 3) 156 mg/kg of HI-6, saline solution dermal; 4) intramuscular saline solution and SM 10 mg/kg; 5) 15.6 mg/kg of HI-6 prior to SM 10 mg/kg; 6) 156 mg/kg of HI-6 prior to SM 10 mg/kg; 7) intramuscular saline solution and SM 40 mg/kg; 8) 15.6 mg/kg of HI-6 prior to SM 40 mg/kg; 9) 156 mg/kg of HI-6 prior to SM 40 mg/kg. The animals were narcotized by carbon dioxide and sacrificed one day after exposure. Blood was collected into a heparinized syringe and processed immediately. After spinning at 1,000×g for ten minutes, plasma and packed (blood) cells were separated. Packed cells were lyzed by addition of four volumes of deionized water. For the anaylsis of DNA damage by comet assay, peripheral lymphocytes were isolated from blood using the gradient centrifugation on lymphoprep (PAA, Austria).

### Ex vivo assays

Ferric reducing antioxidant power (FRAP) and thiobarbituric acid reactive substances (TBARS) were assessed as reported earlier (Pohanka *et al.*, 2011c). 2,4,6-tris(2-pyridyl)-S-triazine, ferric chloride (Sigma-Aldrich; Saint Louis; Missouri; USA) were purchased for FRAP while dimethylsulfoxide, trichloroacetic acid and thiobarbituric acid (Sigma-Aldrich) for TBARS. Glutathione reductase (GR) activity and reduced glutathione (GSH) level in plasma were measured in compliance with the protocol published previously (Pohanka *et al.*, 2011a). Oxidized glutathione, NADPH and EDTA respective 5,5′dithiobis (2-nitrobenzoic acid) and trichloroacetic acid (Sigma-Aldrich) were purchased for GR respective GSH assay purposes.

### Comet assay

Tha alkaline version of the comet assay was used basically as described earlier (Collins *et al.*, 2004). Briefly, lymphocytes embedded in agarose on microscope slides were lysed in 10 mM Tris-buffered 2.5 M NaCl (pH 10.0) containing 1% Triton X 100, 100 mM EDTA for 1 h at 4 °C. In parallel slides, endonuclease III sensitive sites were measured by incubating slides containing lysed nucleoids with endonuclease III (kindly provided by Dr. Karel Angelis, Inst. Exp. Botany, Prague) for 45 min at 37 °C. Then a 40-min period of unwinding was followed by electrophoresis carried out at 25 V, 300 mA, for 30 min at 4 °C. The formed comets (50 cells per slide) were analyzed after staining with ethidiumbromide (Sigma) by comet module of Lucia G image analysis (Laboratory Imaging, Prague, Czech Republic).

### DNA inter-strand cross links (CL)

The amount of CL was measured by modified comet assay. To 1 ml of lymphocyte suspension in RPMI medium (10^6^ cells/ml) styrene oxide (Fluka) was added to the concentration of 600µM and incubated for 30 min at 37°. This induced breaks in DNA leading to the formation of comets containing about 70% DNA in the comet tail. When the DNA contains CL, the alkaline unwinding is blocked and the p.c. of tail DNA is reduced accordingly.

### Statistics

Origin 8 SR2 (OriginLab Corporation, Northampton, MA, USA) was used for data processing and statistical testing. Significance was tested for *p<*0.05 and 0.01 (n=6). One-way ANOVA with Scheffe′s test was used throughout.

The statistical significance of differences in p.c.tail DNA among groups treated with SM and HI-6 were tested by Kruskal-Wallis test and the difference between the group without HI-6 and the HI-6-treated groups were re-tested by Mann-Whitney U-test.

## Results

FRAP level in packed blood cells and plasma was 70–85 mmol/l and 0.6–0.75 mmol/l, respectively. We did not find any significant difference in FRAP values on the tested probability levels and the values for individual groups were overlapped within their deviations. The insignificance was confirmed by a repeated assay one day later. The experimental data are not depicted.

**Figure 1:**
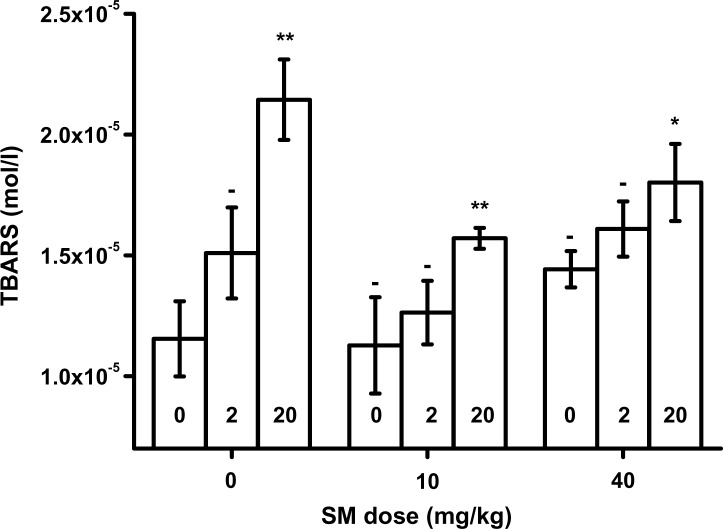
Thiobarbituric acid reacitve substances (TBARS) in blood. Numbers inside bars indicate dose of HI-6 in percent of LD_50_. Error bars were achieved as standard deviations. Probability testing: - none, *p<0.05, **p<0.01. Probability was calculated against group exposed to saline only (no SM and HI-6) for animals exposed to SM only or HI-6 only. Animals exposed to SM and HI-6 were compared to animals exposed to the animals poisoned by the same dose of SM and no HI-6.

**Figure 2:**
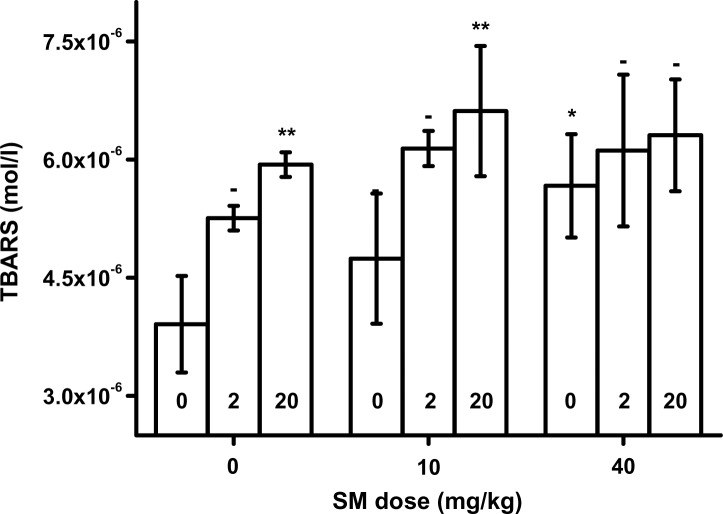
Thiobarbituric acid reacitve substances (TBARS) in plasma. Error bars were achieved as standard deviations. Probability testing: - none, *p<0.05, **p<0.01. Probability was calculated against group exposed to saline only (no SM and HI-6) for animals exposed to SM only or HI-6 only. Animals exposed to SM and HI-6 were compared to animals exposed to the animals poisoned by the same dose of SM and no HI-6.

TBARS value was assessed in blood and plasma. The experimental data are given in Figure 1 for blood and Figure 2 for plasma. For blood samples, TBARS level was slightly elevated with increased SM dose without any co-application of HI-6. Plasma TBARS was significantly elevated in animals exposed to SM in the dose of 40 mg/kg b.w. Co-application of HI-6 and SM or application of HI-6 alone without SM increased TBARS level. The impact of HI-6 was greater in blood than in plasma.

**Figure 3:**
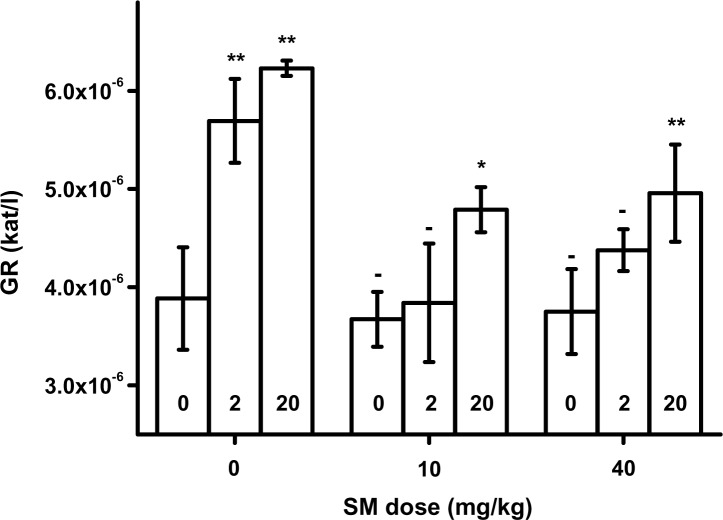
Glutathione reductase (GR) in blood. Error bars were achieved as standard deviations. Probability testing: - none, *p<0.05, **p<0.01. Probability was calculated against group exposed to saline only (no SM and HI-6) for animals exposed to SM only or HI-6 only. Animals exposed to SM and HI-6 were compared to animals exposed to the animals poisoned by the same dose of SM and no HI-6.

**Figure 4:**
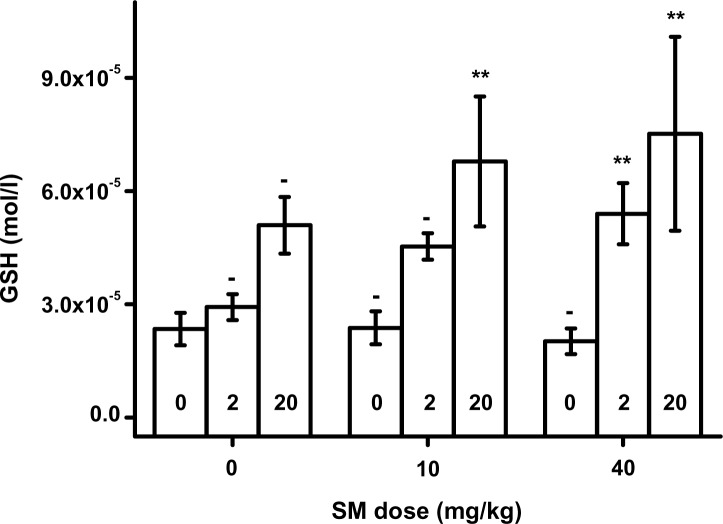
Reduced glutathione (GSH) in plasma. Error bars were achieved as standard deviations. Probability testing: - none, *p<0.05, **p<0.01. Probability was calculated against group exposed to saline only (no SM and HI-6) for animals exposed to SM only or HI-6 only. Animals exposed to SM and HI-6 were compared to animals exposed to the animals poisoned by the same dose of SM and no HI-6.

GR in packed blood is depicted in Figure 3. It was significantly (*p<*0.01) elevated due to HI-6 administration in animals without co-exposition to SM. A similar dose-dependent effect of HI-6 was seen in animals exposed to SM. The effect was however milder and significant (*p=*0.05 for SM 10 mg/kg and *p=*0.01 for SM 40 mg/kg) for the upper doses of HI-6. The plasma level of GSH is presented in Figure 4. A dose-response relationship was found for HI-6. GSH was significantly elevated in animals exposed to SM and the upper doses of HI-6.

**Figure 5:**
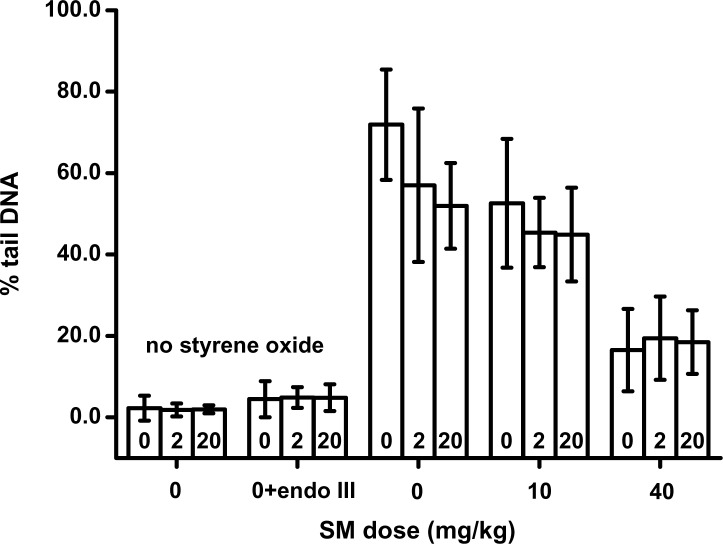
The DNA damage in lymphocytes. Using comet assay the amount of DNA breaks was estimated in isolated lymphocytes of control animals and animals treated with HI-6 only (no styrene oxide). In animals treated simultaneously with HI-6 and SM the lymhocytes were treated with 600 µM to induce DNA breaks and then the comet assay was performed. Mean numbers of 6 animals ± SD are shown.

The relative amount of CL measured in peripheral lymphocytes of animals treated both with HI-6 and SM is shown in Figure 5. As evident from the graph, 2 hours after percutaneous application of HD there was a decrease in the alkaline unwinding of the DNA. The DNA of lymphocytes isolated from control animals not treated with HD unwound after the incubation of cells with 600 µM styrene oxide and about 70% of the DNA migrated into the comet tail during electrophoresis. The migration to the tail was reduced in cells isolated from SM-treated animals in a dose dependent manner to 62 and 18% tail DNA in animals treated with 10 and 40 µM, respectively. This decrease of the alkaline DNA unwinding represents the CL formation in the DNA by SM. As seen in this graph, the application of 2 or 20% LD 50 of HI-6 did not influence CL formation by SM. In cells isolated from control animals with no treatment with SM, we found only small, insignificant induction of oxidized bases detected by endonuclease III (not treated with styrene oxide).

## Discussion

The obtained experimental data show the implication of HI-6 in oxidative stress regulation in the laboratory animal organism. First, we recognized elevated TBARS level in HI-6 treated animals. TBARS represents a biochemical marker of malondialdehyde. Production of malondialdehyde becomes elevated in the organism after oxidative stress initiated lipid peroxidation (Catala, 2011). GR is another marker of oxidative stress. This enzyme is expressed when the oxidative insult exceeds the threshold value (Bogavac *et al.*, 2012). The TBARS levels in blood corresponded with our expectation that HI-6 was implicated in oxidative stress generation (Pohanka, 2011, 2012). GRs levels in HI-6 exposed animals confirmed the previous statement. Regarding the scored GM and TBARS levels, SM had no significant effect on oxidative damage of biological membranes in blood of Wistar rats two hours post poisoning.

The relative amount of DNA cross links (CL) found in lymphocytes of exposed animals clearly show that the doses of HD applied percutaneously induced substantial and significant amount of IC. The findings confirmed that the changes of parameters of oxidative stress presented in this paper resulted from the relatively high intoxication with HD, as reflected in the amount of CL found in peripheral lymphocytes present in the central compartment.

TBARS as well as GR were elevated in animals co-exposed with SM and HI-6. On considering TBARS only, it should be concluded that HI-6 had a negative effect on oxidative stress. On the other hand, a slight oxidative insult could challenge the antioxidant barrier, as proved here for the GR level in blood. Plasmatic GSH had a similar dose-response relationship like blood GR and thus agreement in the two markers increased the relevance of the presented conclusions. The ability of HI-6 to improve the GSH level was also recognized in a previous experiment based on BALB/c mice (Pohanka *et al.*, 2011b). A arise of GSH in brain and kidney was found two hours post exposure. It should be noted that GSH is an important antioxidant playing a privileged role in protection against SM poisoning (Tewari-Singh *et al.*, 2011). From this point of view, HI-6 ameliorated the protection against SM generated oxidative stress even despite, or maybe because, it causes a small oxidative insult. SM is a radiomimetic (Bhattacharya *et al.*, 2001).

The SM toxicity pathway tightly relates to oxidative stress and application of antioxidants is considered a convenient way how to provide protection against detrimental effects of poisoning. The protection of laboratory animals against SM by using antioxidants has been repeatedly recognized (Gautam *et al.*, 2007; 2010; Pohanka *et al.*, 2011c). On the other hand, efficacy of antioxidants is unequal and even EGCG applied in quite high doses failed to abolish oxidative stress in our previous experiment. The main effect of EGCG was rather apoptosis than oxidative stress regulation (Pohanka *et al.*, 2011a). HI-6 has no antioxidant potency, as demonstrated earlier (Pohanka *et al.*, 2011b). Despite this, HI-6 has a plausible ability to trigger the arising enzymatic (GR) and non-enzymatic (GSH) antioxidant accumulation. The molecular mechanism is not clear. There are however some experiments elucidating HI-6 action. It was recognized not only as a noncompetitive inhibitor of the enzyme acetylcholinesterase (Pohanka, 2011, 2012) but also as a noncompetitive antagonist of acetylcholine receptors (Chen *et al.*, 1996). On considering the poor ability of HI-6 to cross biological barriers (Wagner *et al.*, 2010), the main HI-6 action is limited to the blood system. We suggest an implication of HI-6 in the cholinergic system. Owing to its limited ability to penetrate barriers, blood and the peripheral nervous system, represented mainly by the parasympathetic nervous system, can be expected to be the main HI-6 object of action. The cholinergic anti-inflammatory pathway may be the most relevant part of this action (Oke & Tracey, 2009; Pohanka, 2011, 2012). HI-6 showed the potency to antagonize acetylcholine receptors, and in this manner to provide protection from eliciting the parasympathetic cholinergic system, including metabolism and immune response. In light of the obtained experimental data, HI-6 deserves a more complex investigation as a promising non-specific antidote.
